# Salvage chemotherapy with high-dose leucovorin (LV) and 48-hour continuous infusion (CI) of 5-fluorouracil (5-FU) in combination with conventional doses of cyclophosphamide (CPM) in patients with metastatic breast cancer (MBC) pretreated with anthracycline and taxanes

**DOI:** 10.1054/bjoc.2001.2001

**Published:** 2001-09

**Authors:** K Kalbakis, Ch Kouroussis, S Kakolyris, D Mavroudis, J Souglakos, S Agelaki, L Vamvakas, M Christodoulakis, K Stylianou, V Georgoulias

**Affiliations:** Department of Medical Oncology, School of Medicine, University General Hospital of Heraklion,, P.O. Box 1352, Heraklion, Crete, 71110, Greece; Department Surgery Oncology, School of Medicine, University General Hospital of Heraklion, P.O. Box 1352, Heraklion, Crete, 71110, Greece

**Keywords:** breast cancer, salvage chemotherapy, cyclophosphamide, 5-fluorouracil, leucovorin, anthracycline/taxane resistance

## Abstract

The purpose of this study was to evaluate the activity and tolerance of high-dose leucovorin (LV) and infusional 5-fluorouracil (5-FU) in combination with conventional doses of cyclophosphamide (CPM) as salvage chemotherapy in patients with metastatic breast cancer (MBC) pretreated with anthracyclines and taxanes. 41 patients (median age 59 years) with MBC refractory or resistant to anthracyclines and taxanes were enrolled. The patients' performance status (WHO) was 0 in 10 patients (24%), 1 in 22 (54%), and 2 in 9 (22%). 30 (73%) patients had received 2 or more prior chemotherapy regimens. Cyclophosphamide (600 mg m^−2^) was given i.v. bolus on day 1 and LV (500 mg m^−2^ d^−1^) as a 2-h infusion followed by 5-FU (1.5 g m^−2^ d^−1^) over a 22 h c.i. for 2 consecutive days. Cyclophosphamide was administered every 28 days while 5-FU/LV every 14 days. In an intention-to-treat analysis, complete response (CR) was achieved in 2 (4.9%) patients and partial response (PR) in 9 (22%) (overall response rate 26.9%; 95% CI: 13.27–40.39%). Stable disease (SD) and progressive disease (PD) were observed in 9 (22%) and 21 (51%) patients, respectively. The overall response rate was 6% and 40% in patients with primary and secondary resistance to anthracyclines/taxanes, respectively (*P* = 0.047). The median duration of response and the median time to disease progression was 8 and 9.5 months, respectively. The median overall survival was 13 months and the probability for 1-year survival 51%. Grade 3/4 neutropenia occurred in 9 (22%) patients and 4 (9%) patients developed grade 3/4 thrombocytopenia. Non-haematological toxicity was mild. There were no cases of febrile neutropenia, toxic deaths or treatment-related hospital admissions due to toxicity. The combination of high-dose 5-FU/LV with conventional doses of cyclophosphamide is a well tolerated and effective salvage regimen in patients with MBC heavily pretreated with both anthracyclines and taxanes. © 2001 Cancer Research Campaignhttp://www.bjcancer.comhttp://www.bjcancer.com


British Journal of Cancer (2001) **85**(6), 798–802
© 2001 Cancer Research Campaign
doi: 10.1054/ bjoc.2001.2001, available online at http://www.idealibrary.com on IDEAL

http://www.bjcancer.com

### Salvage chemotherapy with high-dose leucovorin (LV) and 48-hour continuous infusion (CI) of 5-fluorouracil (5-FU) in combination with conventional doses of cyclophosphamide (CPM) in patients with metastatic breast cancer (MBC) pretreated with anthracycline and taxanes

**K Kalbakis[1], Ch Kouroussis[1], S Kakolyris[1], D Mavroudis[1], J Souglakos[1], S Agelaki[1], L Vamvakas[1], M Christodoulakis[2], K Stylianou[1] and V Georgoulias[1]**

[1]Department of Medical Oncology, School of Medicine, University General Hospital of Heraklion, P.O. Box 1352, 71110, Heraklion, Crete, Greece; [2]Department Surgery Oncology, School of Medicine, University General Hospital of Heraklion, P.O. Box 1352, 71110, Heraklion, Crete, Greece

**Summary** The purpose of this study was to evaluate the activity and tolerance of high-dose leucovorin (LV) and infusional 5-fluorouracil (5-FU) in combination with conventional doses of cyclophosphamide (CPM) as salvage chemotherapy in patients with metastatic breast cancer (MBC) pretreated with anthracyclines and taxanes. 41 patients (median age 59 years) with MBC refractory or resistant to anthracyclines and taxanes were enrolled. The patients' performance status (WHO) was 0 in 10 patients (24%), 1 in 22 (54%), and 2 in 9 (22%). 30 (73%) patients had received 2 or more prior chemotherapy regimens. Cyclophosphamide (600 mg $m^{-2}$) was given i.v. bolus on day 1 and LV (500 mg $m^{-2}$ $d^{-1}$) as a 2-h infusion followed by 5-FU (1.5 g $m^{-2}$ $d^{-1}$) over a 22 h c.i. for 2 consecutive days. Cyclophosphamide was administered every 28 days while 5-FU/LV every 14 days. In an intention-to-treat analysis, complete response (CR) was achieved in 2 (4.9%) patients and partial response (PR) in 9 (22%) (overall response rate 26.9%; 95% CI: 13.27–40.39%). Stable disease (SD) and progressive disease (PD) were observed in 9 (22%) and 21 (51%) patients, respectively. The overall response rate was 6% and 40% in patients with primary and secondary resistance to anthracyclines/taxanes, respectively ($P$ = 0.047). The median duration of response and the median time to disease progression was 8 and 9.5 months, respectively. The median overall survival was 13 months and the probability for 1-year survival 51%. Grade 3/4 neutropenia occurred in 9 (22%) patients and 4 (9%) patients developed grade 3/4 thrombocytopenia. Non-haematological toxicity was mild. There were no cases of febrile neutropenia, toxic deaths or treatment-related hospital admissions due to toxicity. The combination of high-dose 5-FU/LV with conventional doses of cyclophosphamide is a well tolerated and effective salvage regimen in patients with MBC heavily pretreated with both anthracyclines and taxanes. © 2001 Cancer Research Campaign http://www.bjcancer.com

**Keywords**: breast cancer; salvage chemotherapy; cyclophosphamide; 5-fluorouracil; leucovorin; anthracycline/taxane resistance

Anthracycline- and taxane-based chemotherapy regimens are active as front- or second-line treatment options in patients with MBC resulting in high response rates (Gianni et al, 1995; Ravdin et al, 1995; Conte et al, 1997; Mavroudis et al, 1999). For patients who have failed to respond or relapse early after the taxane/anthracycline regimens, the prognosis is poor since few drugs are still active in this setting (Porkka et al, 1994).

Until the development of new active anticancer agents for the treatment of refractory MBC, we are compelled to use the available agents with the most appropriate manner. Recently, there has been an increasing interest in the treatment of MBC with continuous infusion (c.i.) of 5-fluorouracil (5-FU). Phase II studies of c.i. 5-FU, conducted mainly in heavily pretreated patients, have demonstrated an objective response rate ranging from 12% to 54%, and a median duration of response up to 6 months (Lokich et al, 1989; Cameron et al, 1994). Interestingly, the prolonged infusion of 5-FU was associated with reduced myelotoxicity, which permits the administration of higher doses of the drug. Nevertheless, this mode of administration is accompanied by an increased incidence of stomatitis and palmar-plantar erythrodysaesthesia. This therapeutic benefit with c.i. of 5-FU has also been shown in patients with metastatic colorectal cancer (Lokich et al, 1989).

*Received 11 January 2001*
*Revised 2 May 2001*
*Accepted 4 July 2001*

*Correspondence to:* K Kalbakis

Cyclophosphamide (CPM) is an active agent against MBC given either as first- (ORR = 34%) or second-line (ORR = 22%) treatment (Piccart et al, 1995). Although the combination of cyclophosphamide and doxorubicin remains a standard front-line regimen in MBC, a recent phase III study showed a higher overall response rate and significantly longer time to disease progression in favour of the docetaxel/doxorubicin combination as compared with the combination of doxorubicin/cyclophosphamide (Nabholtz et al, 1999). Therefore, the number of patients who do not receive cyclophosphamide and 5-FU in the front-line setting will be increasing in the future.

Based on these data, we conducted a phase II study to evaluate the activity and tolerance of high dose c.i. 5-FU/LV in combination with conventional doses of cyclophosphamide as salvage chemotherapy in MBC pre-treated patients with both anthracyclines and taxanes.

### PATIENTS AND METHODS

### Eligibility criteria

Patients with histologically confirmed metastatic breast cancer were enrolled. All patients had to have either progression during, or within 6 months of completing anthracyclines and taxanes. In case of no response during the therapy with these agents, the patients were deemed to have primary resistance while in case of objective response for less than 6 months secondary resistance. Patients who had received anthracyclines or taxanes as adjuvant therapy were considered to have resistance to these agents and they were eligible for the study if the disease-free interval was less than 6 months. Other inclusion criteria were: age 18–75 years; performance status (WHO) 0–2; bidimensionally measurable disease; a life expectancy of at least 3 months; adequate hepatic (serum bilirubin ≤ 1.5 times the upper limit of normal; aspartate aminotransferase and alanine aminotransferase ≤ 5.0 times the upper limit of normal) and renal function (serum creatinine ≤ 1.5 mg $dl^{-1}$); absence of active infection or malnutrition; absence of a second primary tumour except of adequately treated in situ carcinoma of the cervix or a non-melanoma skin cancer. Patients with brain metastases were eligible if they had been irradiated, the brain lesions were radiographically stable for at least 2 months post-radiotherapy and clinical improvement was evident. Patients who had received palliation radiotherapy had to have measurable metastatic disease outside the radiation fields. Patients with severe cardiac dysfunction or unstable angina pectoris, or prior irradiation affecting more than 30% of the bone marrow were not eligible. The protocol was approved by the Scientific and Ethics Committee of our Institution and all patients gave written informed consent in order to participate in the study.

### Treatment

Cyclophosphamide was administered on day 1 at the dose of 600 mg $m^{-2}$ in 50 ml normal saline by intravenous (iv) infusion over 15 minutes. Leucovorin (LV) was administered at the dose of 500 mg $m^{-2}$ as a 2-hour i.v. infusion, followed by 5-FU at the dose of 1500 mg $m^{-2}$ as a 22-hours c.i., repeated on 2 consecutive days. Cyclophosphamide was administered every 4 weeks, while 5-FU/LV was administered every 2 weeks. Treatment was continued until disease progression or occurrence of intolerable toxicity. Dose-modification criteria were based on haematological and gastrointestinal toxicity. A 25% dose reduction in all drugs was performed in case of grade 4 granulocytopenia or thrombocytopenia lasting for more than 5 days or febrile neutropenia. In case of ≥ grade 3 diarrhoea, 5-FU/LV doses were reduced by 25% in subsequent cycles. The treatment on day 15 was also postponed for a week if the absolute granulocyte count was <1500 $dl^{-1}$ and the platelet count < 100 000 $dl^{-1}$. No prophylactic administration of growth factors was allowed.

### Evaluation

Pretreatment evaluation included a detailed medical history and physical examination, a complete blood cell count with differential and platelet cell count, whole blood chemistry, determination of serum levels of carcinoembryonic antigen (CEA) and CA 15-3 and computed tomography scans of the chest and abdomen. Additional computed tomography scans and magnetic resonance imaging scans were performed, if clinically indicated. Pretreatment evaluation had to be performed within 2 weeks prior to protocol entry.

During treatment, whole blood counts with differential and platelet counts were performed weekly. A physical examination as well as biochemical tests, determination of serum levels of CEA and CA 15-3 and chest X-rays were performed every 4 weeks. Lesions were evaluated after each cycle if they were assessable by physical examination or by chest X-rays. All patients were assessed by ultrasound and/or computed tomography scans every 3 cycles using the International Union Against Cancer (UICC) criteria for response (Monfardini et al 1987). Toxicity was graded according to the National Cancer Institute common toxicity criteria (Ajani et al, 1990).

### Statistical consideration

This was a 2-step phase II study; if an objective response could be observed in the first 15 patients an additional 15 patients should be enrolled. The duration of response was measured from the first documentation of response to disease progression. The time to tumour progression was determined by the interval between the initiation of treatment and the date of the first documentation of disease progression. The follow-up time was measured from the day of first treatment administration to the last contact or death. The probability of survival was estimated by Kaplan–Meier analysis (Kaplan and Meier, 1959), and the confidence intervals for response rates were calculated using methods for exact binomial confidence intervals (Cox, 1970).

### RESULTS

### Demographics

Between October 1997 and February 2000, 41 pretreated patients with MBC entered the study. Since during the analysis of the first 30 patients the regimen was revealed extremely active, it was decided to enrol 10 additional patients in order to assess more accurately the activity of the regimen. Patients characteristics are shown in Table 1. 32 (78%) patients had a performance status 0–1, and 26 (63%) were postmenopausal. 9 patients (22%) had oestrogen receptors (ER)-positive tumours, 10 (24%) ER-negative, and in 22 (54%) the ER status was unknown. 30 patients (73%) had received 2 or more chemotherapy regimens for the treatment of MBC. 18 (44%) and 15 (37%) patients experienced disease with primary resistance (refractory) to taxanes and anthracyclines, respectively. 20 patients (49%) had 2 or more metastatic sites and 33 (80%) had visceral disease.

### Response to treatment and survival

4 patients were not evaluable for response because they discontinued the treatment before tumour evaluation. The reasons for the early discontinuation of treatment were haemorragic shock due to disseminated intravascular coagulation (one patient), pulmonary embolism (one patient), respiratory insufficiency and metabolic acidosis (one patient) and treatment refusal (one patient). None of

© 2001 Cancer Research Campaign

**Table 1** Patient characteristics

| | No. of patients | % |
|---|---|---|
| Patients enrolled | 41 | |
| Age (years) | | |
| Median | 59 | |
| Range | 39–75 | |
| Performance status (WHO) | | |
| 0 | 10 | 24 |
| 1 | 22 | 54 |
| 2 | 9 | 22 |
| ER status | | |
| Positive | 9 | 22 |
| Negative | 10 | 24 |
| Unknown | 22 | 54 |
| Prior adjuvant chemotherapy | | |
| Anthracycline-based | 10 | 24 |
| Non-anthracycline-based | 31 | 76 |
| Prior regimens for metastatic disease | | |
| 1 | 8 | 20 |
| ≥2 | 33 | 80 |
| Prior treatment with 5-FU/CPM | | |
| 5-FU | 19 | 46 |
| CPM | 18 | 44 |
| Primary resistance | | |
| To taxanes | 18 | 44 |
| To anthracyclines | 15 | 37 |
| Secondary resistance | | |
| To taxanes | 23 | 56 |
| To anthracyclines | 26 | 63 |
| Sites of disease | | |
| Visceral disease | 33 | 80 |
| Non-visceral disease | 8 | 20 |
| No of metastatic sites | | |
| 1 | 21 | 51 |
| ≥2 | 20 | 49 |

these medical complications was felt to be related to the treatment. In an intention-to-treat analysis, 2 (4.9%) patients showed complete response (CR) and 9 (22%) partial response (PR) for an overall response rate of 26.9% (95% C.I.: 13.27–40.39%). Stable disease (SD) and progressive disease (PD) were observed in 9 (22%) and 21 (51%) patients, respectively. All responses were confirmed by repeated CT scans, and/or U/S within 8 weeks after their initial documentation. Responses were observed in all metastatic sites (Table 2) and irrespectively of the treatment line. In patients with primary resistance to anthracyclines and/or taxanes, the overall response rate was 6%, while in patients with secondary resistance 40% ($P = 0.047$). The 2 CRs were observed after 4 and 6 courses of chemotherapy and their duration was 9+ and 6+ months, respectively.

The median duration of response was 8 months (range, 1–20) and the median time to disease progression 9.5 months (range, 4.5–22.5).

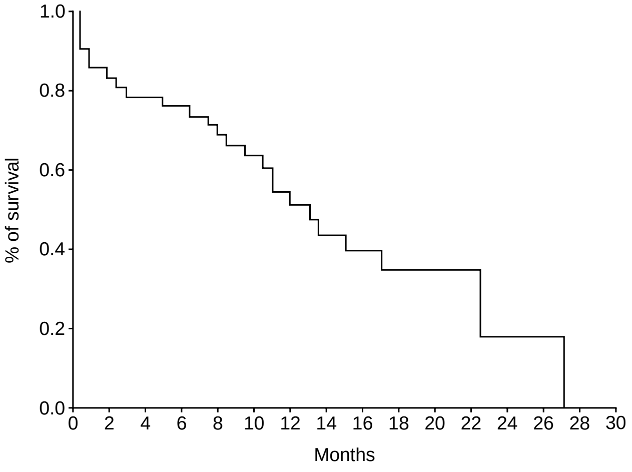

**Figure 1** Kaplan–Meier survival curve

After a median follow-up period of 10.5 months (range, 0.5–27), 25 patients (61%) died; the median overall survival time was 13 months and the probability for 1-year survival 51% (Figure 1).

### Compliance to treatment

A total of 192 chemotherapy courses were administered with a median number of 3 courses per patient (range, 1–18). 13 patients (32%) received more than 6 courses. The median interval between courses was 28 days (range, 28–47), and treatment was delayed in 47 courses (24.5%) corresponding to 15 patients (37%) for the following reasons: haematological toxicity (10 cycles), non-haematological toxicity (2 cycles) and for miscellaneous reasons unrelated to the treatment (35 cycles).

Dose reduction was required in only 4 courses (4%) because of haematological toxicity. The median administered dose intensity of cyclophosphamide was 146 mg $m^{-2}$ $week^{-1}$ (range, 82–150), of 5-FU 1.412 mg $m^{-2}$ $week^{-1}$ (range, 750–1500) and of LV 471 mg $m^{-2}$ $week^{-1}$ (range, 250–500). The relative dose intensity was 94% for cyclophosphamide and 93% for both 5-FU and LV. In 27 patients (66%) treatment was discontinued because of progressive disease. One patient refused further treatment after the first cycle.

### Toxicity

The haematological and non-haematological toxicity of the regimen is presented in Table 3. The haematological toxicity was generally mild with 9 (22%) patients developing grade 3/4

**Table 2** Response according to metastatic site ($n = 37$)

| Site | No. of patients CR | PR | SD | PD |
|---|---|---|---|---|
| Soft tissues ($n = 17$) | 1 (6%) | 5 (29%) | 2 (12%) | 9 (53%) |
| Lymph nodes ($n = 13$) | 1 (8%) | 3 (23%) | – | 9 (69%) |
| Liver ($n = 17$) | – | 5 (29%) | 5 (29%) | 7 (42%) |
| Pleura ($n = 15$) | – | 4 (27%) | 3 (20%) | 8 (53%) |
| Lung ($n = 17$) | 1 (6%) | 3 (18%) | 3 (18%) | 10 (58%) |

 *© 2001 Cancer Research Campaign*

**Table 3** Haematological and non-haematological toxicity of patients treated with CPM+5FU/LV (CI)

| | Number of patients (%) | | | |
|---|---|---|---|---|
| **Toxicity (Grade; NCI)** | **1** | **2** | **3** | **4** |
| Neutropenia | 10 (24) | 7 (17) | 4 (10) | 5 (12) |
| Anaemia | 22 (54) | 10 (24) | 1 (2) | – |
| Thrombocytopenia | 17 (41) | – | 1 (2) | 3 (7) |
| Nausea/vomiting | 12 (29) | 6 (15) | – | – |
| Mucositis | 8 (20) | 2 (5) | – | – |
| Diarrhoea | 4 (10) | 1 (2) | – | – |
| Constipation | 12 (29) | 5 (12) | 1 (2) | – |
| Fatigue | 16 (39) | 13 (32) | 3 (7) | – |
| Neurotoxicity | 4 (10) | – | – | – |
| Skin reactions | 1 (2) | – | – | – |

neutropenia; no patient developed febrile neutropenia and there was no death due to toxicity. 4 (9%) patients presented grade 3/4 thrombocytopenia and all had received prior radiotherapy for metastatic bone lesions in an area covering 25% of their bone marrow. One patient developed grade 3 anaemia. The non-haematological toxicity was mild. Grade 3 fatigue was observed in 3 (7%) patients and grade 3 constipation in one; no other grade 3 or 4 toxicity was observed. No hospital admission was required because of treatment-related complications.

### DISCUSSION

As an increasing number of patients with breast cancer receive anthracyclines and/or taxane-based chemotherapy as adjuvant or front-line treatment, the therapeutic options for these patients when they experience early disease-progression is a difficult task in oncology practice. Moreover, the total dose of anthracyclines which has been administered either in the adjuvant setting or in front-line treatment precludes further administration of this agent in second-line regimens. In addition, taxane-related neurotoxicity also makes difficult their use in the second line. Frequently, these patients have a good performance status and require any effort to improve, at least, the symptoms of their disease and their quality of life.

In this study, by using the combination of continuous infusion of 5-FU and cyclophosphamide, 22% of the patients achieved an objective response and 27% stabilization of their disease. However, it is noteworthy that 80% of the patients had visceral disease and had already received 2 or more chemotherapy regimens based on the most active agents against breast cancer. It is interesting to note that there was a statistically significant difference of the overall response rate in patients with primary (ORR = 6%) and secondary resistance (ORR = 40%; $P = 0.047$), indicating that this combination should not be prescribed in the former group of patients.

The interest in the use of prolonged infusions of 5-FU in the treatment of breast cancer is not new. In 1987, Hansen et al (1987) reported an overall response rate of 32%, with a continuous 5-FU regimen in 5-FU-pretreated patients. The toxicity was significant but tolerable. Similar results have been reported by other investigators (Chang et al, 1989; Jabboury et al, 1989). Many agents have been used in combination with continuous 5-FU. Gordon et al (1990) reported that the combination of protracted infusion of 5-FU with weekly bolus administration of doxorubicin and oral cyclophosphamide was associated with an overall response rate of 82% in patients with metastatic breast cancer irrespective of the prior treatment; however mucositis was the major dose-limiting toxicity. More recently, Dogliotti et al (1999) reported that the combination of vinorelbine with protracted infusion of 5-FU resulted in an overall response rate of 68% (14% CR) in heavily pretreated patients. Most of their patients had failed or relapsed after an anthracycline-based first-line chemotherapy regimen and 43% after a taxane-based second-line treatment. The toxicity was mild as only 2 patients showed grade 4 thrombocytopenia and grade 3 neurotoxicity, each. In another Italian study, the protracted infusion of 5-FU in anthracycline refractory breast cancer patients was associated with a 33% overall response rate and a 6 months median duration of response (Crivellari et al, 1999). The toxicity was mild although grade 3–4 hand-foot syndrome occurred in 27% of the patients. In the same study, the addition of vinorelbine was associated with an enhanced response rate (ORR: 68%), at the price of an increased toxicity; toxicity was mostly non-haematologic as 28% and 21% of the patients showed mucositis and hand-foot syndrome, respectively.

In our study, although the patient selection criteria were different than most of the aforementioned studies, the overall response rate was significantly lower but the median duration of response (8 months) and the median time to disease progression (9.5 months) were encouraging. The 13 months median overall survival is also promising for these poor prognosis patients. On the other hand, the toxicity was mild with no grade 3/4 non-haematological toxicity. It seems that the improved response rates with the protracted infusion of 5-FU, which have been previously reported (Dogliotti et al, 1999), were achieved at the price of increased incidence of mucocutaneous toxicity; however, there was no benefit in terms of median time to disease progression and overall survival. Our findings are in agreement with the phase I/II study, reported by Borguez et al (2000); in this trial the intermittent administration of infusional 5-FU in combination with vinorelbine resulted in a promising antitumour efficacy with grade 3/4 neutropenia occurring in 39% of the cycles but without grade 3 or 4 non-haematological toxicity. Therefore, it seems that the intermittent continuous infusion of 5-FU/LV is better tolerated than the protracted one providing better palliation in heavily pretreated breast cancer patients. In addition, this regimen does not require the continuous use of an infusion pump, as well as the increased expenses related to their use. Although, our patients were hospitalized for 48 hours in order to receive the 5-FU c.i., this could have also been achieved in an out-patient basis using an indwelling catheter and an infusion pump, thus reducing the cost of the treatment. The relative utility of this regimen should also be judged by comparing it with other alternatives such as vinorelbine or capecitabine which do not require hospital admission or infusional pumps. A cost-effective comparison of our regimen with these agents is not possible since there are few available data regarding their activity in this patient population and they are both associated with significant haematological and non-haematological toxicity.

In conclusion, the results of the present study demonstrate that the combination of cyclophosphamide and LV-modulated bolus plus infusional 5-FU is an active and safe salvage chemotherapy regimen especially in patients with MBC who have secondary resistance to anthracyclines and taxanes. However, these data should be interpreted with caution because of the small number of patients included in this study. Additional studies are needed in order to compare the intermittent continuous infusion of 5-FU with the protracted one in terms of efficacy and tolerance.

© 2001 Cancer Research Campaign 

### REFERENCES

Ajani JA, Welch SR, Raber MN, Fields WS and Krakoff IH (1990) Comprehensive criteria for assessing therapy-induced toxicity. *Cancer Invest* **8**: 147–159

Borguez D, Vanhoefer U, Oberhoff C, Hense J, Mayer S, Bojko P, Harstrick A and Seeber S (2000) Phase I/II study of vinorelbine in combination with a weekly schedule of folinic acid and infusional 5-FU in patients with metastatic breast cancer (Abstract 420). *Proc ASCO* **14**: 109a

Cameron DA, Gabra H and Leonard RC (1994) Continuous 5-fluorouracil in the treatment of breast cancer. *Br J Cancer* **70(1)**: 120–124

Chang AY, Most C and Pandya KJ (1989) Continuous intravenous infusion of 5-fluorouracil in the treatment of refractory breast cancer. *Am J Clin Oncol* **12**: 453–455

Conte PF, Baldini E, Gennari A, Michelotti A, Salvadori B, Tibaldi Danesi R, Innocenti F, Gentile A, Dell'Anna R, Biadi O, Mariani M and Del Tacca M (1997) Dose-finding study and pharmacokinetics of epirubicin and paclitaxel over 3 hours: A regimen with high activity and low cardiotoxicity in advanced breast cancer. *J Clin Oncol* **15**: 2510–2517

Cox DR (1970) *The analysis of Binary Data*. Methuen: London

Crivellari D, Magri DM, Buonadonna A, De Cicco M, Ferlante AM, Paolello C and Veronessi A (1999) Palliative treatment with 5-fluorouracil (FU) continuous infusion (ci) ± Navelbine (NVB) in metastatic, anthracycline refractory breast cancer (Abstract 436). *Proc ASCO* **18**: 115a

Dogliotti L, Berruti A, Sperone P, Gorzegno G, Bottini A, Tampellini M, Donadio M and Alquati P (1999) Vinorelbine and protracted infusional 5-fluorouracil is a very active and manageable scheme for heavily pretreated advanced breast cancer patients (Abstract 432). *Proc ASCO* **18**: 114a

Gianni L, Munzone E, Capri G, Fulfaro F, Tarenzi E, Villani F, Spreafico C, Laffranchi A, Caraceni A and Martini C (1995) Paclitaxel by 3 hour infusion in combination with bolus doxorubicin in women with untreated metastatic breast cancer: High antitumor efficacy and cardiac effects in a dose-finding and sequence-finding study. *J Clin Oncol* **13**: 2688–2699

Gordon CJ, Valdivieso M, Martino S, Redman BG, Flaherty L and Baker LH (1990) Continuous intravenous 5-fluorouracil (5-FU), weekly adriamycin (ADR) and oral cyclophosphamide (CTX) (FAC-CT) in the treatment of metastatic breast carcinoma (MBC) (Abstract 200). *Proc ASCO* **9**: 52

Hansen R, Quebbeman E, Beatty P, Ritch P, Anderson T, Jenkins D, Frick J and Ausman R (1987) Continuous 5-fluorouracil in refractory carcinoma of the breast. *Breast Cancer Res Treat* **10**: 145–149

Jabboury K, Holmes FA and Hortobagyi G (1989) 5-fluorouracil rechallenge by protracted infusion in refractory breast cancer. *Cancer* **64**: 793–797

Kaplan EL and Meier P (1959) Non-parametric estimation from incomplete observations. *J Am Stat Assoc* **53**: 457–481

Lokich JJ, Ahlgren JD, Gullo JJ, Phillips JA and Fryer JG (1989) A prospective randomized comparison of continuous infusion fluorouracil with a conventional bolus schedule in metastatic colorectal carcinoma. *A mid-Atlantic Oncology Program Study J Clin Oncol* **7**: 425–432

Mavroudis D, Malamos N, Alexopoulos A, Kouroussis Ch, Agelaki S, Sarra E, Potamianou A, Kosmas C, Rigatos G, Giannakakis T, Kalbakis K, Apostolaki F, Vlachonikolis J, Kakolyris S, Samonis G and Georgoulias V (1999) Salvage chemotherapy in anthracycline-pretreated metastatic breast cancer patients with docetaxel and gemcitabine: A multicenter phase II trial. *Ann Oncol* **10**: 211–215

Monfardini S, Brunner K and Crowther D (1987) Evaluation of the cancer patient and the response to treatment. In UICC-Manual of Adult and Paediatric Medical Oncology (ed.) pp 22–38. Springer 1: Berlin

Nabholtz JM, Falkson G, Campos D, Szanto J, Martin M, Chan S, Pienkowski T, Bezwoda WR, Zaluski J, Pinter T, Krazakowski M, Vorobiof D, Leonard R, Kennedy I, Azli N, Murawsky M, Riva A and Pouillart P (1999) A phase III trial comparing doxorubicin (A) and docetaxel (T) (AT) to doxorubicin and cyclophosphamide (AC) as first line chemotherapy for MBC (Abstract 485). *Proc ASCO* **18**: 127a

Piccart MJ, Raymond E, Aapro M, Eisenhauer EA and Cvitkovic E (1995) Cytotoxic agents with activity in breast cancer patients previously exposed to anthracyclines: Current status and future prospects. *Eur J Cancer* **31A**(7): 1–10

Porkka K, Blomgvist C, Prissamen P, Elomaa I and Pyrhonen S (1994) Salvage therapies in women who fail to respond to first-line treatment with fluorouracil, epirubicin and cyclophosphamide for advanced breast cancer. *J Clin Oncol* **12**(8): 1639–1647

Ravdin PM, Burris HA, Cook G, Eisenberg P, Kane M, Bierma WA, Mortimer J, Genevois E and Bellet RE (1995) Phase II trial of docetaxel in advanced anthracycline-resistant or anthracenedione-resistant breast cancer. *J Clin Oncol* **13**: 2879–2885 1

 *© 2001 Cancer Research Campaign*
